# DHA Shortage Causes the Early Degeneration of Photoreceptors and RPE in Mice With Peroxisomal β-Oxidation Deficiency

**DOI:** 10.1167/iovs.64.14.10

**Published:** 2023-11-07

**Authors:** Daniëlle Swinkels, Sai Kocherlakota, Yannick Das, Adriaan D. Dane, Eric J. M. Wever, Frédéric M. Vaz, Nicolas G. Bazan, Paul P. Van Veldhoven, Myriam Baes

**Affiliations:** 1 Laboratory of Cell Metabolism, Department of Pharmaceutical and Pharmacological Sciences, KU Leuven, Leuven, Belgium; 2 Department of Epidemiology and Data Science, Amsterdam UMC, University of Amsterdam, Amsterdam, The Netherlands; 3 Core Facility Metabolomics, Amsterdam UMC, University of Amsterdam, Amsterdam, The Netherlands; 4 Department of Clinical Chemistry and Pediatrics, Laboratory Genetic Metabolic Diseases, Emma Children's Hospital, Amsterdam UMC, University of Amsterdam, Amsterdam, The Netherlands; 5 Inborn Errors of Metabolism, Amsterdam Gastroenterology Endocrinology Metabolism, Amsterdam, The Netherlands; 6 Neuroscience Center of Excellence, Louisiana State University School of Medicine, Louisiana State University, New Orleans, Louisiana, United States; 7 Laboratory of Peroxisome Biology and Intracellular Communication, Department of Cellular and Molecular Medicine, KU Leuven, Leuven, Belgium

**Keywords:** DHA, VLC-PUFA, peroxisome, RPE, neuroprotection

## Abstract

**Purpose:**

Patients deficient in peroxisomal β-oxidation, which is essential for the synthesis of docosahexaenoic acid (DHA, C22:6n-3) and breakdown of very-long-chain polyunsaturated fatty acids (VLC-PUFAs), both important components of photoreceptor outer segments, develop retinopathy present with retinopathy. The representative mouse model lacking the central enzyme of this pathway, multifunctional protein 2 (*Mfp2^−/−^*), also show early-onset retinal decay and cell-autonomous retinal pigment epithelium (RPE) degeneration, accompanied by reduced plasma and retinal DHA levels. In this study, we investigated whether DHA supplementation can rescue the retinal degeneration of *Mfp2^−/−^* mice.

**Methods:**

*Mfp2^+/−^* breeding pairs and their offspring were fed a 0.12% DHA or control diet during gestation and lactation and until sacrifice. Offspring were analyzed for retinal function via electroretinograms and for lipid composition of neural retina and plasma with lipidome analysis and gas chromatography, respectively, and histologically using retinal sections and RPE flatmounts at the ages of 4, 8, and 16 weeks.

**Results:**

DHA supplementation to *Mfp2^−/−^* mice restored retinal DHA levels and prevented photoreceptor shortening, death, and impaired functioning until 8 weeks. In addition, rescue of retinal DHA levels temporarily improved the ability of the RPE to phagocytose outer segments and delayed the RPE dedifferentiation. However, despite the initial rescue of retinal integrity, DHA supplementation could not prevent retinal degeneration at 16 weeks.

**Conclusions:**

We reveal that the shortage of a systemic supply of DHA is pivotal for the early retinal degeneration in *Mfp2^−/−^* mice. Furthermore, we report that adequate retinal DHA levels are essential not only for photoreceptors but also for RPE homeostasis.

The fatty acid composition of photoreceptor outer segment (POS) phospholipids is peculiar, as it is highly enriched in polyunsaturated fatty acids (PUFAs).^1^ The most abundant PUFA in the POS, which can amount to 50% of the phospholipid side chains, is the omega-3 fatty acid docosahexaenoic acid (DHA, C22:6n-3),^2^ but also substantial amounts of very-long-chain PUFAs (VLC-PUFAs, >28 carbons) occur.^3^ Both DHA and VLC-PUFAs are involved in several crucial processes in photoreceptors, underscoring their importance.^4^

Recently, the metabolism and trafficking of these lipids were extensively reviewed.^4^ In short, the DHA content in the body originates from dietary sources, containing either the mature form or its precursor α-linolenic acid (ALA, 18:3n-3).^2^^,^^5^ The synthesis of DHA from ALA involves elongations, desaturations, and a retroconversion that is executed by one cycle of peroxisomal β-oxidation, also known as the Sprecher pathway.^5^^–^^7^ This process mostly takes place in the liver^8^ but can also occur in photoreceptors and the retinal pigment epithelium (RPE).^9^^–^^12^ Plasma DHA reaches the photoreceptor inner segments (PISs) after transport through the RPE and is esterified into phospholipids required for POS biogenesis. Alternatively, DHA can be further elongated to VLC-PUFAs, which is, among others, catalyzed by the elongase of very-long-chain fatty acid 4 (ELOVL4).^13^ During the daily phagocytosis, the lipid-rich POSs are taken up by the RPE and its components are either degraded or recycled back to the PISs.^14^

Interestingly, peroxisomal β-oxidation is involved in several aspects of the metabolism of (VLC-)PUFAs. In addition to its role in the synthesis of DHA, it is also involved in the breakdown of DHA and VLC-PUFAs.^15^ Regarding the high abundance of peroxisomal β-oxidation enzymes in the different retinal cells,^16^^,^^17^ these metabolic processes are presumed to take place in both the photoreceptors and RPE. The importance of peroxisomal β-oxidation in the retina is supported by the fact that patients with peroxisomal β-oxidation deficiency present with retinopathy.^18^^–^^20^ Unfortunately, data on histological^21^^,^^22^ and lipid changes^23^ in the retina of patients with a deficiency in peroxisomes are scarce.

We recently studied the mechanism underlying the retinal degeneration in peroxisome deficiency, by analyzing a mouse model lacking the central enzyme of peroxisomal β-oxidation, multifunctional protein 2 (MFP2).^24^ These *Mfp2^−/−^* mice presented with both developmental and degenerative anomalies, including (1) POS shortening at 2 weeks, (2) progressive photoreceptor degeneration, (3) impaired visual function (3 weeks),^24^ and (4) RPE dedifferentiation and lysosomal dysfunction (3 weeks).^25^ Furthermore, transcriptome analysis on neural retina samples revealed drastic changes in pathways related to phototransduction, photoreceptor-specific transcription factors, lipid metabolism, inflammation, and cell death already at the age of 3 weeks.^24^ Moreover, DHA-containing phospholipid species were severely depleted in both the retina and plasma of *Mfp2^−/−^* mice.^24^ In contrast, mice lacking MFP2 specifically in photoreceptors (*Crx-Mfp2^−/−^* mice) displayed intact retinal DHA levels and photoreceptor development.^26^ These findings hint at the importance of the systemic supply of DHA for retinal DHA levels and integrity in *Mfp2^−/−^* mice.

Therefore, we aimed to restore the systemic supply of DHA in *Mfp2^−/−^* mice by supplementing a 0.12% (w/w) DHA diet. This treatment replenished retinal DHA levels in the *Mfp2^−/−^* retina and prevented photoreceptor shortening, death, and impaired functioning at early ages. Surprisingly, DHA supplementation also improved RPE morphology and functioning. However, improvements in photoreceptor and RPE homeostasis were only temporary, regardless of continuous DHA supplementation, indicating that DHA supplementation alone will not be sufficient to prevent vision loss in MFP2-deficient patients. Altogether, these data provide a better understanding of the synthesis, trafficking, and function of DHA in the retina and shed new light on the pathogenesis of the retinopathy in peroxisome-deficient patients.

## Materials and Methods

### Mouse Handling

Global *Mfp2* knockout mice (Swiss background) were generated by breeding heterozygous mice.^27^ As no retinal differences were observed between *Mfp2^+/+^* and *Mfp2^+/−^* mice, both served as control. Genotyping for *Mfp2^−/−^* and the spontaneously occurring *rd1* mutation (*Pde6* gene) was performed as described previously (see Supplementary Table S1).^24^^,^^28^

Animals were bred in the conventional animal housing facility of the KU Leuven and were kept on a 13-hour/11-hour light/dark cycle. Experiments conformed to the ARVO Statement for the Use of Animals in Ophthalmic and Vision Research and were approved by the Research Ethical Committee of the KU Leuven (P166/2017 and P129/2022). Mice were sedated with a mix of Nimatek (75 mg/kg) and Domitor (1 mg/kg). To collect plasma, the eye was removed, followed by collection of blood in heparin pretreated tubes and centrifugation at 1200*g* for 10 minutes at 4°C. The neural retina and RPE were isolated as previously explained, 7 hours after light onset.^24^^,^^25^^,^^29^

### Dietary Intervention

Swiss *Mfp2^+/−^* pregnant female mice and their offspring were fed a 0.12% DHA or control diet (Supplementary Fig. S1). The dietary DHA supplements (TG-form; MEG3) were kindly provided by DSM Nutritional Products (Basel, Switzerland) and incorporated into the chow at ssniff Spezialdiäten (Soest, Germany). The concentration and composition of the DHA diet were based on Connor et al.^30^ and consisted of 9.1% safflower oil and 0.9% MEG3. The control diet contained 10% safflower oil.

To determine the fatty acid composition of the diets, they were extracted via the Bligh–Dyer method and analyzed by gas chromatography (GC).^31^^,^^32^ To this end, the diet was finely ground, dissolved in methanol/chloroform (2:1 v/v; 1 g diet/3 mL solvent), and the liquid phase was collected. Next, 1-M NaCl/methanol (7:3 v/v) was added, the upper phase was removed, while the lower phase was dried in Genevac EZ-2 (Sysmex, La Hulpe, Belgium) and sent for GC analysis.

The fatty acids in the control diet mainly consisted of palmitic acid (C16:0), oleic acid (C18:1n-9), and linoleic acid (C18:2n-6) (Table), whereas DHA (C22:6n-3) could not be detected. The DHA diet was comprised of the same main lipid species as the control diet but was partly substituted with 1.2% (w/w of total fatty acids) DHA. As the diets contained a total of 10% fat, the DHA diet consisted of 0.12% (w/w) DHA.

**Table.** Fatty Acid Composition of Control, DHA, and Standard Diet (% w/w of Total Fatty Acids)^*^

Lipids	Control Diet	DHA Diet	Standard Chow
C16:0	6.1	7.5	19.4
C18:0	n.d.	n.d.	4.4
C18:1n-9	74.0	70.2	14.2
C18:2n-6	17.1	15.8	54.6
C18:3n-3 (ALA)	0.1	0.2	6.1
C18:3n-6	n.d.	n.d.	0.1
C20:0	0.4	0.4	0.2
C20:4n-6 (AA)	n.d.	0.1	n.d.
C20:5n-3 (EPA)	0.8	1.7	0.2
C22:0	0.4	0.4	0.3
C22:4n-6	n.d.	n.d.	n.d.
C22:5n-3 (DPA)	n.d.	n.d.	n.d.
C22:5n-6	n.d.	n.d.	n.d.
C22:6n-3 (DHA)	n.d.	1.2	n.d.
C24:0	0.2	0.2	0.2

AA, arachidonic acid; ALA, α-linolenic acid; DHA, docosahexaenoic acid; DPA, docosapentaenoic acid; EPA, eicosapentaenoic acid; n.d., not detected.

* Only the relevant fatty acids are shown.

### Electroretinogram

To measure visual functionality, electroretinograms (ERGs) were performed using the Celeris system (Diagnosys, Lowell, MA, USA), in collaboration with the laboratory of Animal Physiology and Neurobiology, KU Leuven.^26^

### Lipid Measurements

To determine the total fatty acids, GC analysis was performed by the Amsterdam UMC as previously defined.^33^ After correction using an internal standard, fatty acid levels were expressed as a percentage of total fatty acids (diet) or µmol/L extract (plasma).

Lipidome analysis on *Mfp2^−/−^* neural retina samples was performed by the Core Facility Metabolomics of the Amsterdam UMC, as described previously.^26^^,^^34^ Of note, comparisons could only be performed between different groups (e.g., wild-type (WT) vs. *Mfp2^−/−^*) within the same lipid classes. Data are presented as fold change compared to WT levels on the control diet.

### Histological Assessments

Enucleated eyes were fixed overnight at 4°C in new Davidson's fixative (NDF; 22.2% [v/v] formaldehyde 10%, 32% [v/v] alcohol, 11.1% [v/v] glacial acetic acid), after which they were cut into 7-µm-thick transverse retinal sections. Gross morphology was assessed with hematoxylin and eosin (H&E) staining.^26^ Based on Lobanova et al.,^35^ the number of photoreceptor nuclei were counted over a distance of 100 µm at six different regions on both sides (nasal and temporal) of the optic nerve head, after which the number of nuclei were summed up. Images were acquired with an inverted IX-81 microscope (20× objective; Olympus, Tokyo, Japan). To measure the length of the photoreceptor layer (PR), POS, and PIS, phase-contrast microscopy was performed as previously explained.^26^ Images were acquired with a Leica DMI6000 B microscope, using phase-contrast settings (63× objective; Leica, Wetzlar, Germany).

Immunohistochemical (IHC) analysis was performed on NDF sections and RPE flatmounts as described.^26^ Primary antibodies are listed in Supplementary Table S2. Images were acquired with a Leica SP8 confocal microscope. To calculate the number of rhodopsin-positive POSs over a distance of 100 µm, per mouse one image was taken on either side of the optic nerve (100× objective), after which POSs were counted using ImageJ (National Institutes of Health, Bethesda, MD, USA), and the average was used for analysis.^36^ Of note, the rhodopsin B630 variant was used, which recognizes the N-terminus of rhodopsin that remains intact until fusion with and degradation in lysosomes.^37^

### Immunoblotting

Neural retinas were homogenized as described before.^26^ RPEs were homogenized using a pestle homogenizer for 30 seconds, after which the sclera was discarded. Next, immunoblotting was performed using the described protocol,^26^ with the exception of the blocking buffer (5% [w/v] bovine serum albumin in 0.1% [v/v] Tween 20). Primary antibodies are listed in Supplementary Table S2. Images were processed with Image Lab software (Bio-Rad, Hercules, CA, USA). Vinculin served as loading control.

### RNA Isolation and Quantitative RT-PCR

The neural retina was homogenized in TRIzol (Thermo Fisher Scientific, Waltham, MA, USA) with a sonicator. The RPE was homogenized in lysis buffer (PureLink RNA Mini Kit; Thermo Fisher Scientific) containing 2-mercaptoethanol using a pestle homogenizer for 30 seconds, after which supernatants were collected. Next, RNA was extracted and converted to cDNA, and quantitative RT-PCR (RT-qPCR) was performed as previously described.^24^ To calculate the relative expression to a reference gene (*Actb*), the 2^−ΔΔCT^ method was used. Primers are listed in Supplementary Table S3.

### Statistics and Reproducibility

Statistical analysis was performed using Prism 9.3 (GraphPad, San Diego, CA, USA). Grubbs’ test was executed on every dataset to identify possible outliers, the Shapiro–Wilk test was used to assess normal distribution, and the *F*-test was performed to test equality of the variances. Statistical significance was set at *P* < 0.05, and data are presented as mean ± SD. Two-way ANOVA was used to analyze ERG responses, and one-way ANOVA with multiple comparison was performed for the other tests.

## Results

### Supplementing a DHA Diet to *Mfp2^−/−^* Mice Normalizes Plasma and Neural Retina DHA Levels

Our previous findings of lowered plasma and retinal DHA levels in *Mfp2^−/−^* mice^24^ encouraged us to investigate whether levels could be restored by supplementing DHA to the diet. To this end, a 0.12% (w/w) DHA or control diet was fed to *Mfp2^+/^^−^* pregnant female mice and their offspring until sacrifice (Supplementary Fig. S1). Of note, previous studies describing the retinal phenotype of *Mfp2^−/−^* mice were performed on C57Bl6 mice,^24^ whereas these supplementation studies were done on Swiss *Mfp2^−/−^* mice because of the larger litters.

**Figure 1.**
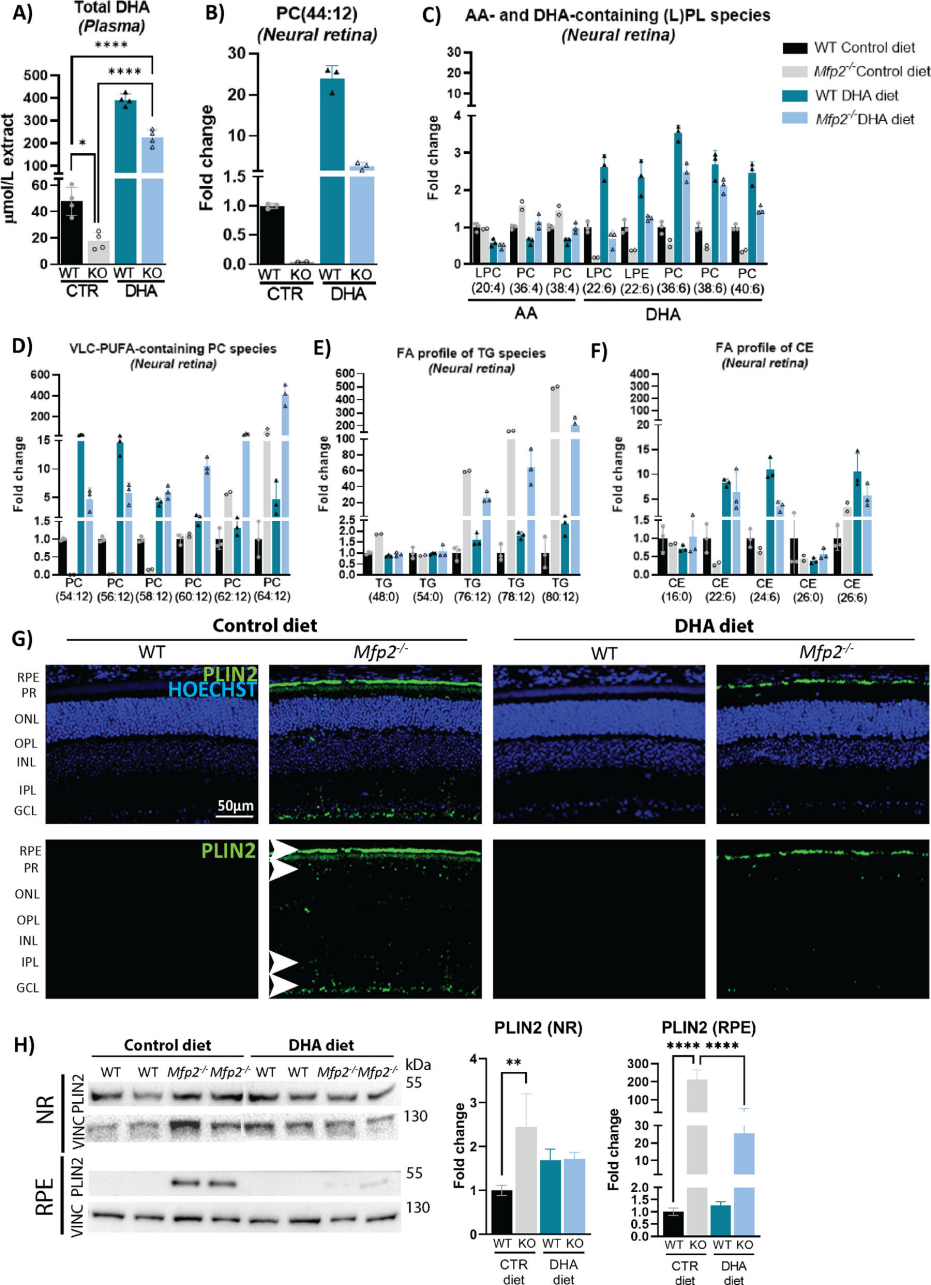
Altered lipid profile in DHA-supplemented *Mfp2^−/−^* mice (4 weeks). (**A**) GC analysis to determine DHA levels in plasma (*n* = 4/group). (**B**, **C**) Lipidome analysis represented as fold change for (lyso-)phospholipids, (L)PLs most likely containing two DHA moieties (**B**) or one DHA or AA moiety (**C**). (**D**) VLC-PUFA–containing phospholipid species. (**E**, **F**) Fatty acid profile of triglycerides (**E**) and cholesteryl esters (**F**) (*n* = 2 or 3/group). No statistical test was performed for the lipidome data, but individual data points are shown. (**G**) IHC staining for the lipid droplet marker PLIN2 (*green*) on 4-week-old mice. The *lower panel* serves to only visualize the lipid droplets. (**H**) Immunoblotting and quantification of PLIN2 levels in 4-week-old neural retina and RPE. Vinculin was used as loading control (*n* = 4/group). Statistical differences for plasma DHA levels and immunoblotting were based on multiple one-way ANOVA. *Error bars* indicate SD. RPE, retinal pigment epithelium; PR, photoreceptor; ONL, outer nuclear layer; OPL, outer plexiform layer; INL, inner nuclear layer; IPL, inner plexiform layer; GCL, ganglion cell layer; PC, phosphatidylcholine; PL, phospholipids; TG, triglycerides; DHA, docosahexaenoic acid; AA, arachidonic acid; CE, cholesteryl esters; VLC-PUFA, very-long-chain polyunsaturated fatty acid; PLIN2, perilipin 2; NR, neural retina; VINC, vinculin. *P < 0.05; **P < 0.01; ****P < 0.0001.

First, we assessed DHA levels in plasma of 4-week-old mice by GC analysis. As expected, DHA supplementation to *Mfp2^−/−^* mice increased plasma DHA, which even rose above levels in WT mice on the control diet (fourfold) (Fig. 1A). Notably, plasma DHA reached almost twofold higher levels in WT mice on the DHA diet than in *Mfp2^−/−^* mice on the same diet. Other plasma lipid species are provided in Supplementary Table S4.

Subsequently, we evaluated the effect of DHA supplementation via an extensive lipidome analysis on 4-week-old neural retinas, as DHA is primarily localized to the POS. Examination of glycerophospholipid species such as PC(44:12), which is generally accepted to contain two DHA moieties, revealed a 2.7-fold increase in the neural retina of *Mfp2^−/−^* mice on the DHA diet compared to levels of WT mice on the control diet (Fig. 1B). Furthermore, other phospholipid species and cholesteryl esters most likely containing DHA (based on the presence of six or 12 double bonds) increased similarly (Figs. 1C, 1F; Supplementary Fig. S2).

Another interesting finding was that, in contrast to *Mfp2^−/−^* mice on the control diet, there was no compensatory upregulation of the omega-6 fatty acid arachidonic acid (C20:4n-6) in the DHA-supplemented *Mfp2^−/−^* retina (Fig. 1C). Due to technical limitations during the COVID-19 pandemic, only two or three replicates could be used for lipidomic analysis, restricting the possibility of performing statistical tests. Nevertheless, the data appear to be representative, as (1) there is low variation between the replicates, and (2) the lipid changes in the *Mfp2^−/−^* retinas on the control diet are consistent with previously reported changes in C57Bl6 *Mfp2^−/−^* mice.^24^

The phosphatidylcholine (PC) phospholipids were further analyzed with regard to incorporation of VLC-PUFAs, as those were previously reported to show a peculiar profile in the neural retina of C57Bl6 *Mfp2^−/−^* mice.^24^ PC species, most likely composed of one DHA and one VLC-PUFA moiety, up to a length of 36 carbons (e.g., PC[58:12]), were almost absent in the neural retina of *Mfp2^−/−^* mice on the control diet (80%–90% reduced) but reached levels above normal in the DHA-supplemented *Mfp2^−/−^* mice (5- to 10-fold increase) compared to WT mice on the control diet (Fig. 1D). This implies that the reduced VLC-PUFA levels (≤C36) in the *Mfp2^−/−^* retina were due to the lack of the precursor DHA. In contrast, PC species containing longer VLC-PUFAs (≥C38), such as PC[60:12], accumulated in *Mfp2^−/−^* mice on either diet, but this was more pronounced in the DHA- supplemented *Mfp2^−/−^* mice, likely due to uncontrolled PUFA elongation.

Considering that VLC-PUFAs can also be incorporated into other lipid species in the neural retina,^26^ the PUFA composition of triglycerides (TGs) was examined. TG species presumably containing one common saturated fatty acid (e.g., C16:0, C18:0), one DHA moiety (C22:6), and one VLC-PUFA elongated from DHA (e.g., C38:6, C40:6, C42:6) accumulated to a lesser extent (two- to threefold) in the neural retina of *Mfp2^−/−^* mice on the DHA diet compared to *Mfp2^−/−^* mice on the control diet (Fig. 1E). Nevertheless, levels of these TG species were still increased by 20- to 200-fold compared to WT mice on the control diet. On the other hand, levels of TG species containing saturated fatty acids, such as three times C16:0 (i.e., TG[48:0]) or three times C18:0 (TG[54:0]) were unaltered. Also the composition of the other neutral lipid species able to store VLC-PUFAs, i.e. cholesteryl esters, were analyzed. However, no drastic increases in VLC-PUFA–containing CE species were found (Fig. 1F), most likely because the storage of fatty acids in CE species is less important in the neural retina compared to the RPE.^38^

Although VLC-PUFAs are enriched in the photoreceptors, it should be kept in mind that the levels of PUFAs (≥C24) are several orders of magnitude lower than long-chain saturated or monounsaturated fatty acids.^39^ However, our analysis does not allow us to compare the absolute concentrations of different lipid species given the semiquantitative nature of the measurement. Therefore, we evaluated if the altered composition of the neutral lipids in the neural retina impacted on the abundance of lipid droplets by performing IHC staining for the lipid droplet marker perilipin-2 (PLIN2) at 4 weeks. Although no lipid droplets were detected in WT mice on both diets, PLIN2 staining was clearly present in the photoreceptors, inner plexiform, and ganglion cell layer of the neural retina of *Mfp2^−/−^* mice on the control diet (Fig. 1G). Interestingly, DHA supplementation reduced the neutral lipid accumulation in the entire neural retina of *Mfp2^−/−^* mice (Fig. 1G) compared to *Mfp2^−/−^* mice on the control diet. This was quantified using immunoblotting for PLIN2, revealing normalization of PLIN2 in the neural retina of DHA-supplemented *Mfp2^−/−^* mice compared to WT mice (Fig. 1H).

The PLIN2 staining also showed extensive lipid droplet accumulation in the *Mfp2^−/−^* RPE, similar to previous findings in C57Bl6 mice.^25^ Remarkably, similar to the neural retina, DHA supplementation considerably reduced the number of lipid droplets in the RPE, as shown by PLIN2 IHC and immunoblotting (90% reduction) (Figs. 1G, 1H).

Overall, the lipid analyses revealed that supplementing a 0.12% (w/w) DHA diet to *Mfp2^−/−^* mice enhanced the systemic supply of DHA to the retina, thereby restoring levels of DHA-containing phospholipid species in the neural retina. This also affected the levels and distribution of the elongation products. However, the lipidomics data need to be carefully interpreted, as no statistical tests were performed.

### Normalizing Retinal DHA Levels Improves Photoreceptor Development and Delays Retinal Degeneration in *Mfp2^−/−^* Mice

**Figure 2.**
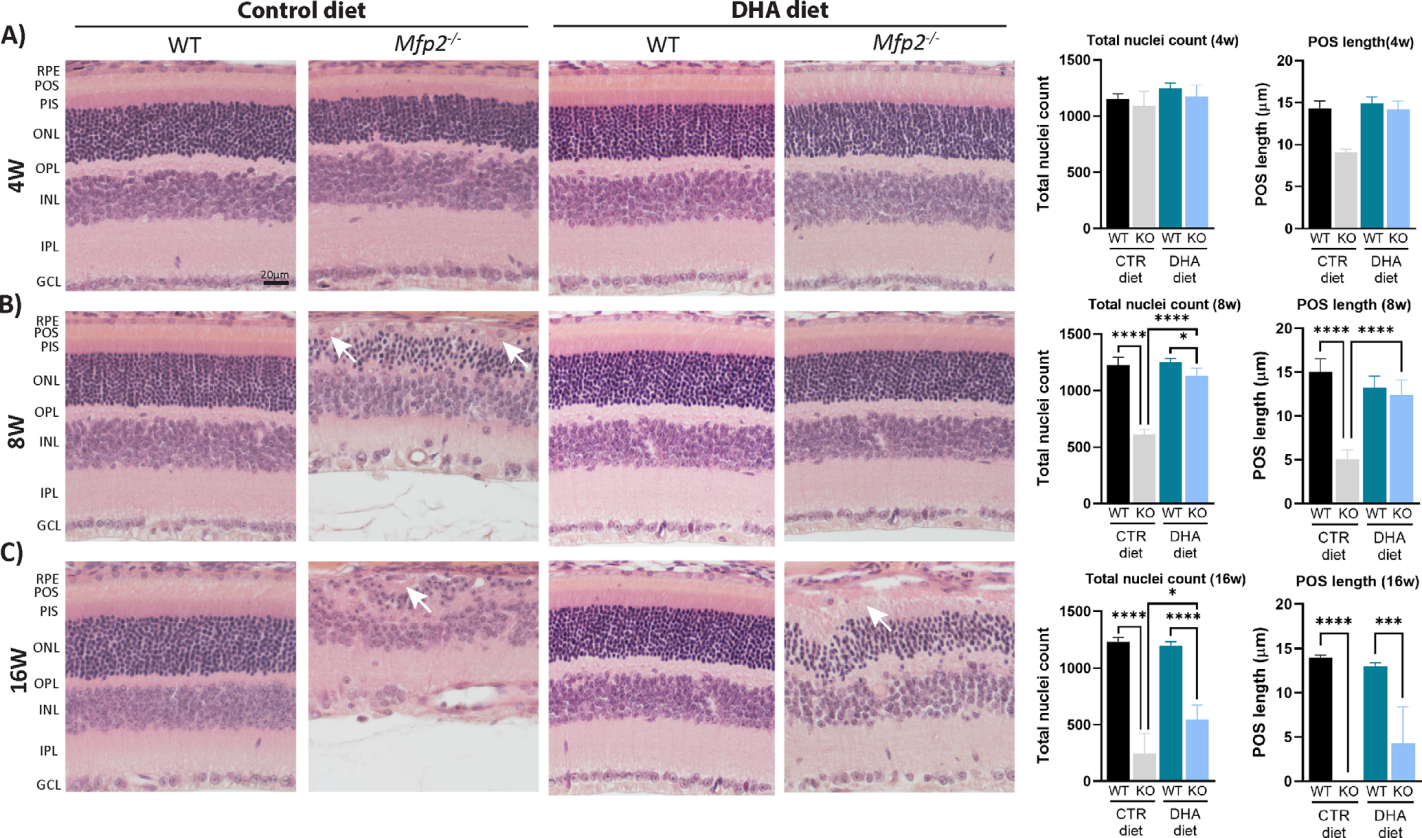
DHA supplementation to *Mfp2^−/−^* mice delays retinal degeneration. (**A**–**C**) H&E stainings are shown for 4-week-old (**A**), 8-week-old (**B**), and 16-week-old (**C**) mice. The *right panel* represents quantification of the ONL nuclei count and POS length per respective age. *White arrows* indicate dedifferentiated RPE cells (*n* = 4 or 5/group). Statistical differences are based on multiple one-way ANOVA. *Error bars* indicate SD. RPE, retinal pigment epithelium; POS, photoreceptor outer segment; PIS, photoreceptor inner segment; ONL, outer nuclear layer; OPL, outer plexiform layer; INL, inner nuclear layer; IPL, inner plexiform layer; GCL, ganglion cell layer; CTR, control. *P < 0.05; ***P < 0.001; ****P < 0.0001.

*Mfp2^−/−^* mice in the C57Bl6 background presented with POS shortening already at the age of 2 weeks and loss of photoreceptors at 8 weeks.^24^ To explore whether restoring DHA levels in the *Mfp2^−/−^* retina could prevent these retinal abnormalities, H&E stainings (Fig. 2) and morphometric analysis (Supplementary Fig. S3) on retinas of 4-, 8-, and 16-week-old DHA-supplemented *Mfp2^−/−^* mice were performed. Interestingly, although *Mfp2^−/−^* mice on the control diet presented with 30% shorter POSs at 4 weeks, this was not observed in *Mfp2^−/−^* mice on the DHA diet (Fig. 2A, Supplementary Fig. S3A). Even more striking were the findings at 8 weeks. The severe shortening (60%) and loss of photoreceptors (50%) in *Mfp2^−/−^* mice on the control diet were prevented by supplementation with DHA (Fig. 2B, Supplementary Fig. S3B). However, despite the initial rescue of retinal integrity, DHA supplementation could not prevent a substantial retinal degeneration at 16 weeks. Nevertheless, POS length and photoreceptor survival were still better preserved compared to *Mfp2^−/−^* mice on the control diet at the same age (Fig. 2C, Supplementary Fig. S3C). Of note, no differences in retinal morphology were observed between WT mice on either diet (Figs. 2A–2C, quantifications). Taken together, these results indicate that impaired systemic delivery of DHA to the neural retina caused the early-onset photoreceptor developmental and degeneration problems in *Mfp2^−/−^* mice. Nevertheless, other mechanisms are also at play, as the retina deteriorated at 16 weeks, despite continuous DHA supplementation.

### Supplementation of DHA Improves *Mfp2^−/−^* Visual Function

**Figure 3.**
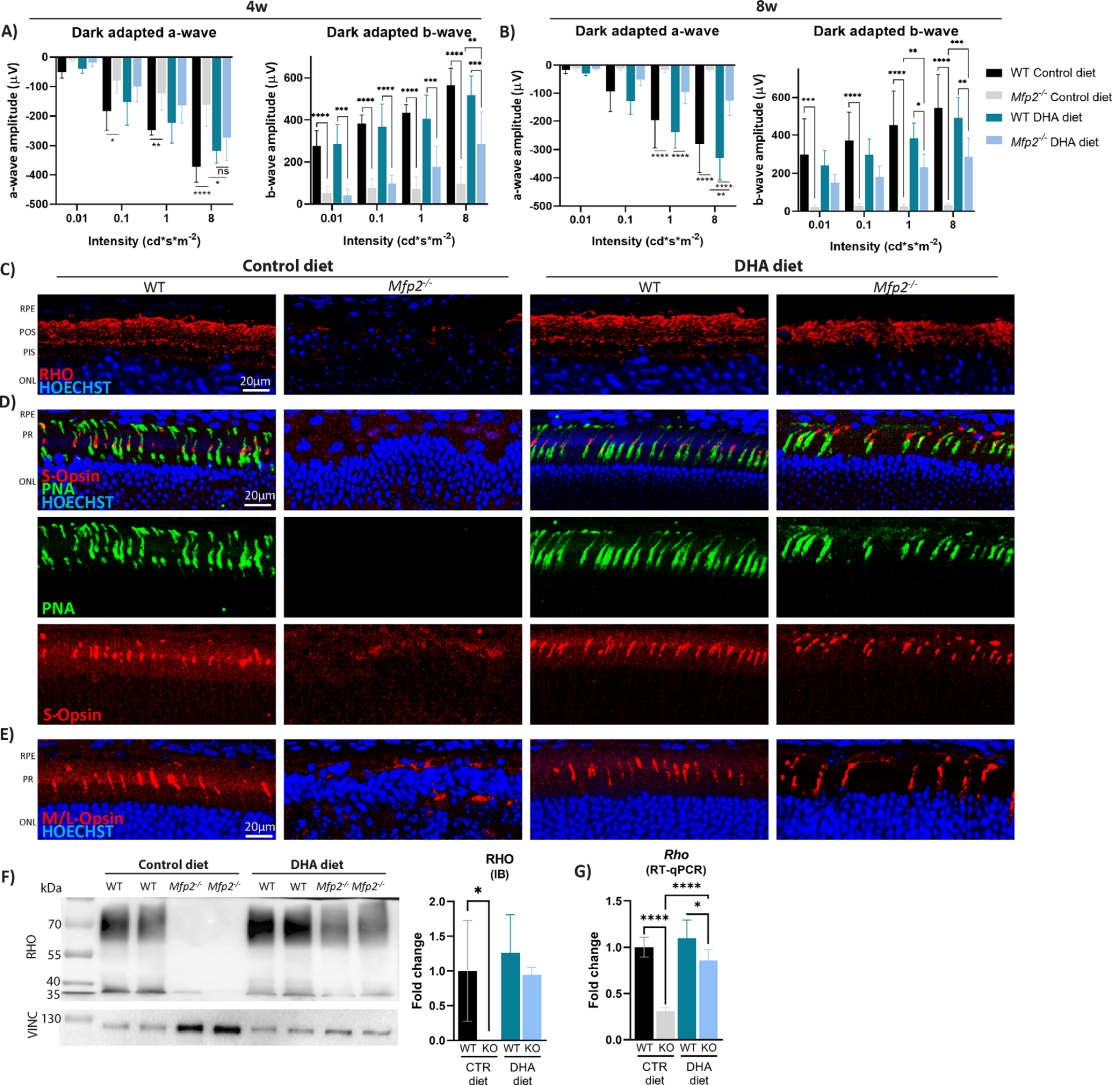
DHA supplementation improves photoreceptor functioning and morphology in *Mfp2^−/−^* mice. (**A**, **B**) Dark-adapted ERG responses from 4-week-old mice (**A**) and 8-week-old mice (**B**). (**C**) Rod-specific stainings visualized with rhodopsin (*red*). (**D**, **E**) Cone-specific stainings on 8-week-old retinal sections visualized with S-opsin (*red*) and peanut agglutinin lectin (PNA; *green*) (**D**) and M/L-opsin (*red*) (**E**). (**F**) Immunoblotting (IB) and quantification of rhodopsin protein (8 weeks). (**G**) RT-qPCR for rhodopsin mRNA levels (8 weeks) (*n* = 4–8/group). Statistical differences are based on multiple two-way ANOVA for the ERGs and one-way ANOVA for immunoblotting and RT-qPCR. *Error bars* indicate SD. RPE, retinal pigment epithelium; POS, photoreceptor outer segments; PIS, photoreceptor inner segments; ONL, outer nuclear layer; OPL, outer plexiform layer; RHO, rhodopsin; PNA, peanut agglutinin lectin; VINC, vinculin; CTR, control. *P < 0.05; **P < 0.01; ***P < 0.001; ****P < 0.0001.

Next, we evaluated if restored retinal DHA levels and photoreceptor integrity in juvenile *Mfp2^−/−^* mice impacted on the function of rods, the dominant photoreceptors in the mouse retina, by measuring ERGs. Scotopic (i.e., dark-adapted) a-wave responses, which represent the activity of rod photoreceptors, of 4-week-old *Mfp2^−/−^* mice on the control diet were reduced (Fig. 3A), similar to previous observations in knockouts in the C57Bl6 background.^24^ DHA supplementation normalized the rod photoreceptor response at the highest intensity in 4-week-old *Mfp2^−/−^* mice, which is in accordance with the intact morphology of these neurons at this age (Figs. 2A, 3A). However, already at 8 weeks, the a-wave response deteriorated, despite normal retinal morphology at this age, although responses were still ±fourfold higher than in *Mfp2^−/−^* mice on the control diet (Fig. 3B). The scotopic b-wave response, which represents the activity of rod interneurons, was almost absent in 4- and 8-week-old *Mfp2^−/−^* mice on the control diet (Figs. 3A, 3B). The DHA diet improved the interneuron response, but it could not normalize them to WT levels. These findings are in line with our previous observations in *Crx-Mfp2^−/−^* mice—that is, normal photoreceptor responses but impaired interneuron responses.^26^

To investigate the underlying reason for the declined rod photoreceptor responses at 8 weeks, the localization and levels of the rod photoreceptor-specific marker rhodopsin were assessed in 8-week-old mice. In line with the H&E stainings (Fig. 2B), IHC staining showed an impressive improvement in rod photoreceptor length and survival in DHA-supplemented *Mfp2^−/−^* mice compared to *Mfp2^−/−^* mice on the control diet (Fig. 3C). This was accompanied by normalization of rhodopsin protein and even transcript levels (Figs. 3F, 3G).

Also the function and integrity of the cone photoreceptors were assessed in DHA-supplemented *Mfp2^−/−^* mice. However, there was a high variability within the groups for the ERG responses, most likely because (1) the mouse retina is rod dominant, making it difficult to reliably measure cone-driven responses; and (2) non-pigmented mice were used in this study, which have been shown before to have lower cone-driven ERG responses compared to pigmented mice.^40^ Indeed, only a trend to reduction was observed in the cone photoreceptor responses in *Mfp2^−/−^* mice on the control diet versus WT mice on the same diet (4 and 8 weeks) (Supplementary Figs. S4A, S4B). Interestingly, DHA-supplemented *Mfp2^−/−^* mice did not show this trend to reduction in both 4- and 8-week-old mice, and cone-driven responses were similar to WT mice on the same diet (Supplementary Figs. S4A, S4B). In addition, the integrity of the different cone photoreceptors (short (S), medium (M), and long (L)) was assessed. Noteworthy, both the morphology and survival of the different cone photoreceptors markedly improved over the entire retina in *Mfp2^−/−^* mice due to DHA supplementation (8 weeks) (Figs. 3D, 3E; Supplementary Figs. S4C, S4D). Overall, these findings reinforce previous reports that normal retinal DHA levels are essential for photoreceptor morphology and functioning.^41^^–^^49^

### DHA Supplementation Delays RPE Dedifferentiation in *Mfp2^−/−^* Mice

The RPE exerts an array of functions, with their main goal being to maintain photoreceptor health. Accordingly, dysfunctions of the RPE cause degeneration of the retina. This was recently shown for mice lacking MFP2 specifically in the RPE (*Best1-Mfp2^−/−^* mice).^25^ These mice presented with early-onset RPE dedifferentiation similar to that of global *Mfp2^−/−^* mice. This consisted of loss of hexagonal shape, RPE depolarization, ablation of visual cycle proteins, and RPE protrusions, causing secondary retinal degeneration at later ages.^25^ Therefore, it was of interest to investigate whether increased levels of DHA in the retina impacted on the RPE dedifferentiation process.

**Figure 4.**
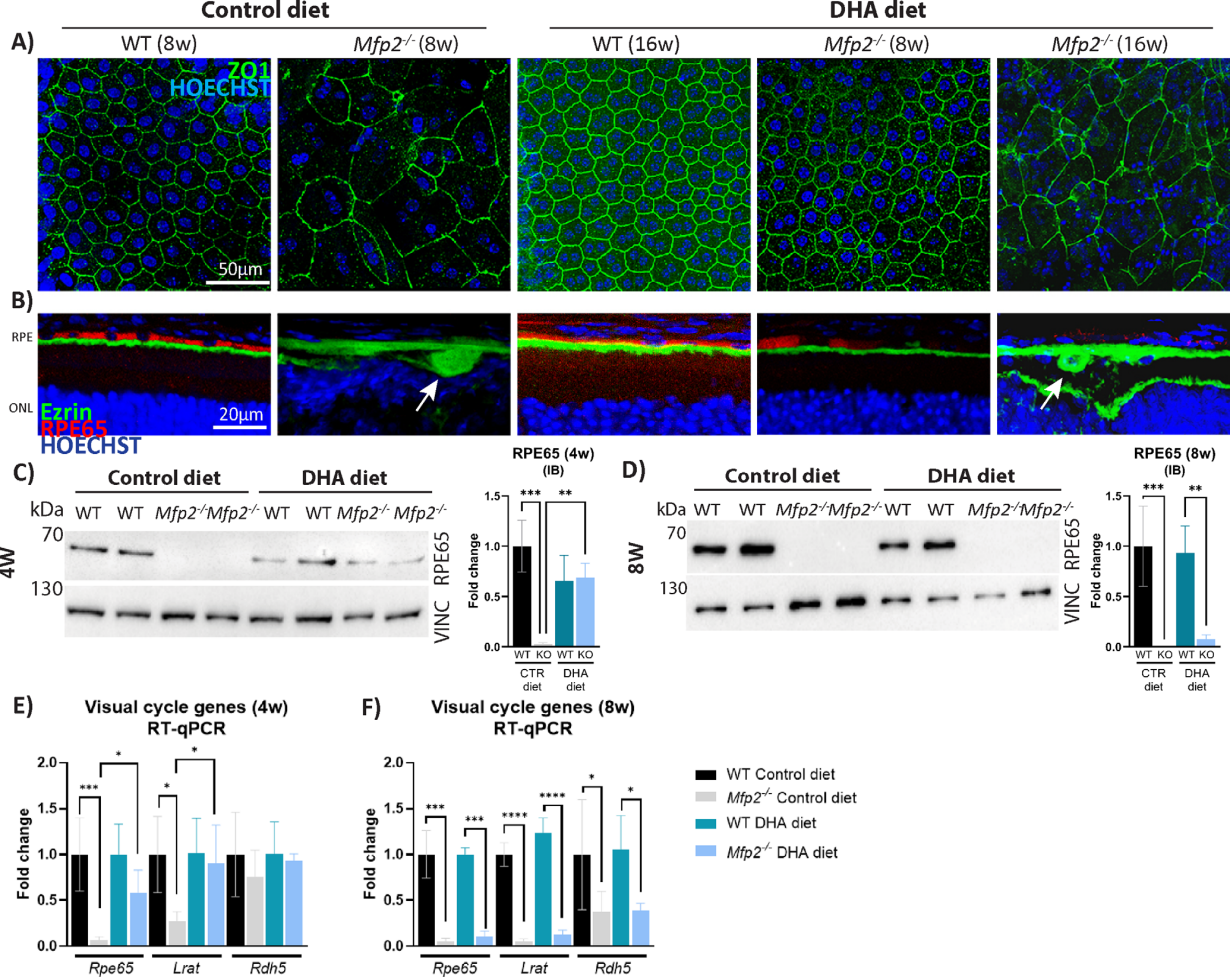
DHA supplementation delays RPE dedifferentiation in *Mfp2^−/−^* mice. (**A**) ZO1 staining (*green*) on RPE wholemounts of 8- and 16-week-old mice. (**B**) IHC double staining for RPE65 (*red*) and ezrin (*green*) on 8- and 16-week-old mice. *White arrows* indicate RPE protrusions. (**C**, **D**) Immunoblotting (IB) for RPE65 in 4-week-old (**C**) and 8-week-old (**D**) RPE samples. (**E**, **F**) RT-qPCR for the visual cycle genes at 4 weeks (**E**) and 8 weeks (**F**) (*n* = 4–7/group). Statistical differences are based on multiple one-way ANOVA. *Error bars* indicate SD. LRAT, lecithin retinol acyltransferase; RDH5, retinol dehydrogenase 5; RPE, retinal pigment epithelium; ONL, outer nuclear layer; ZO1, zonula occludens-1; RPE65, 65-kDa retinoid isomerohydrolase; VINC, vinculin; CTR, control; *P < 0.05; **P < 0.01; ***P < 0.001; ****P < 0.001.

Strikingly, whereas the RPE of 8-week-old *Mfp2^−/−^* mice on the control diet was strongly distorted, RPE cells maintained their hexagonal shape upon DHA supplementation, visualized with the tight junction marker zonula occludens protein 1 (ZO1) (Fig. 4A). Furthermore, staining for ezrin, an apical marker that mislocalized to the basolateral side in *Mfp2^−/−^* mice on the control diet, remained localized to the apical side in *Mfp2^−/−^* mice on the DHA diet (Fig. 4B). In addition, no RPE protrusions into the POS layer were seen (8 weeks) (Figs. 2B, 4B, white arrows). Moreover, transcript and protein levels of the crucial visual cycle protein 65-kDa retinoid isomerohydrolase (RPE65) were normal in the RPE of 4-week-old *Mfp2^−/−^* mice on the DHA diet (Figs. 4C, 4E). This was also the case for other visual cycle genes (Fig. 4E). However, in contrast to the other RPE features, at 8 weeks the visual cycle genes were suppressed by more than 60% to 90% compared to WT mice, despite DHA supplementation (Fig. 4F). This coincided with a patchy signal for RPE65 in the RPE and severe loss of RPE65 protein, visualized with IHC and immunoblotting, respectively (8 weeks) (Figs. 4B, 4D). Remarkably, the changes in visual cycle genes were not associated with alterations in the transcription factors Sox9 or Otx2, which regulate their expression (Supplementary Fig. S5).^50^ At 16 weeks, the RPE dedifferentiated (including loss of hexagonal shape, RPE depolarization, and RPE protrusions), despite continuous DHA supplementation (Figs. 2C, 4A, 4B). These data indicate that DHA is important in maintaining RPE differentiation but that other mechanisms are also involved.

### Supplementation of DHA Affects the Lysosomal Functioning of the *Mfp2^−/−^* RPE

Another essential function of the RPE is the daily phagocytosis of damaged POSs. Importantly, we recently demonstrated that loss of peroxisomal β-oxidation in the RPE impairs the digestive function of lysosomes, whereby the degradation of both POS phagosomes and the autophagic cargo was hampered. The prolonged presence of undigested POSs caused prolonged activation of mammalian target of rapamycin (mTOR), which is known to trigger RPE dedifferentiation.^25^ Hence, it was of interest to evaluate if DHA supplementation influenced the lysosomal function of the *Mfp2^−/−^* RPE.

**Figure 5.**
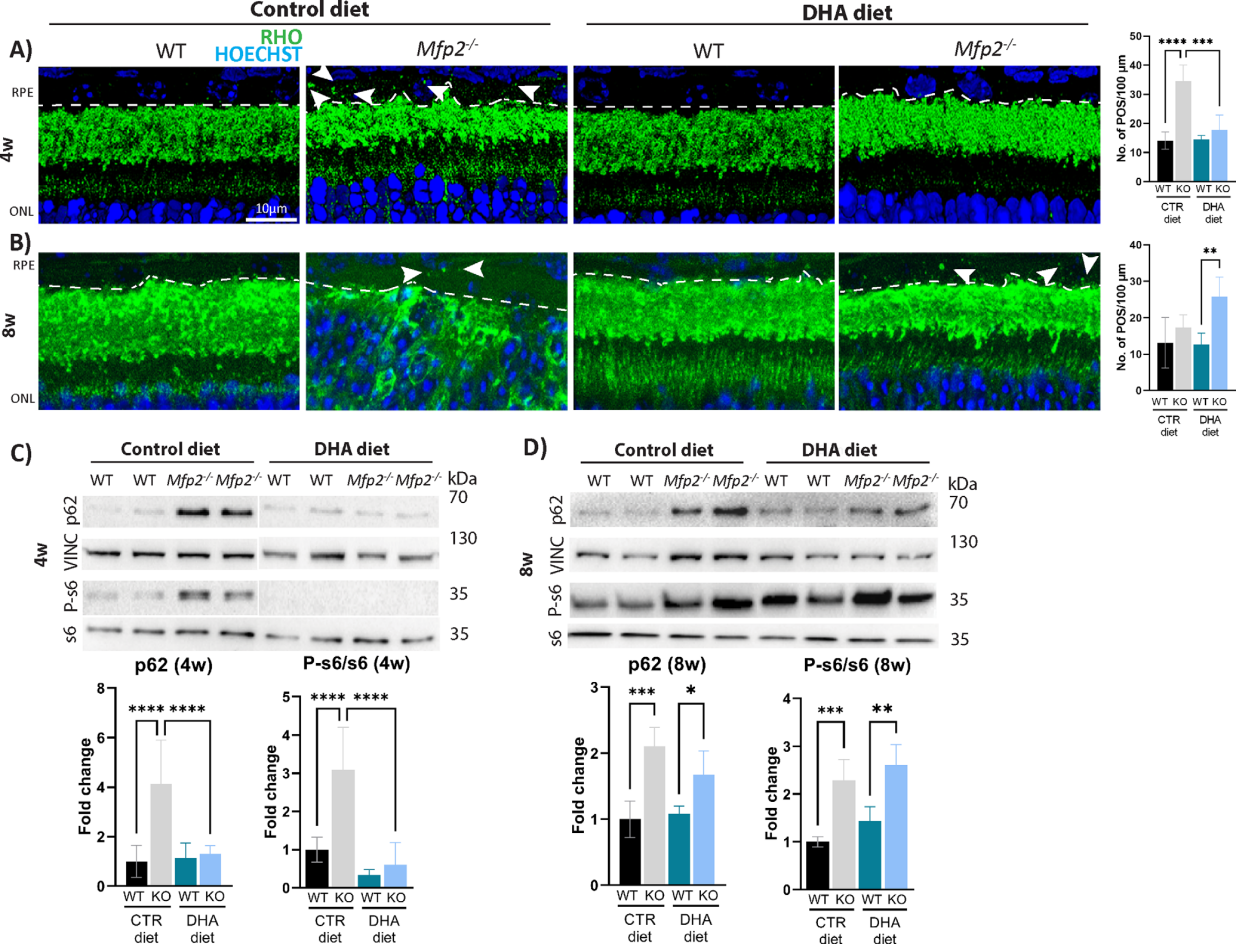
Improved RPE functioning in 4-week-old DHA-supplemented *Mfp2^−/−^* mice. (**A**, **B**) IHC staining on 4-week-old (**A**) and 8-week-old (**B**) mice for rhodopsin-containing POS particles (*green*) (illustrative instances are indicated with *white arrows*) in the RPE (delineated with *dotted line*). Quantifications of rhodopsin-positive POS per 100 µm are located on the *right*, per respective age. (**C**, **D**) Immunoblotting for p62, P-s6, and s6 on 4-week-old (**C**) and 8-week-old (**D**) RPE samples. Vinculin was used as loading control (*n* = 4–8/group). Statistical differences are based on multiple one-way ANOVA. *Error bars* indicate SD. RPE, retinal pigment epithelium; ONL, outer nuclear layer; RHO, rhodopsin; P-s6, phosphorylated ribosomal protein s6; VINC, vinculin; CTR, control; *P < 0.05; **P < 0.01; ***P < 0.001; ****P < 0.0001.

Characteristic of dysfunctional lysosomes in the RPE is the accumulation of rhodopsin-containing POS particles,^36^ which we previously demonstrated to accrue in C57Bl6 mice.^25^ Similarly, more rhodopsin-containing POS particles were observed in the RPE of 4-week-old *Mfp2^−/−^* mice on the control diet (Fig. 5A). Intriguingly, this accumulation was not observed in the *Mfp2^−/−^* RPE supplemented with DHA at the same age, indicating the improved ability of the RPE to digest the POS (Fig. 5A). Next, the status of mTOR was evaluated, by assessing levels of the phosphorylated form of its downstream target s6 (P-s6). In line with the notion that prolonged mTOR activation is caused by undigested POSs, P-s6 levels were not upregulated in the RPE of *Mfp2^−/−^* mice on the DHA diet (Fig. 5C). Finally, the lysosomal degradation of the autophagic cargo was evaluated, by measuring levels of p62, a protein that binds and marks the autophagic cargo for degradation. Indeed, although p62 vastly accumulated in the RPE of *Mfp2^−/−^* mice on the control diet (fourfold), this did not occur in *Mfp2^−/−^* mice on the DHA diet at 4 weeks (Fig. 5C). These findings indicate that DHA supplementation rescues the lysosomal functioning of the *Mfp2^−/−^* RPE at 4 weeks. However, the rescue of lysosomal functioning was only temporary, as 8-week-old *Mfp2^−/−^* RPE cells supplemented with the DHA diet showed POS accumulation, prolonged activity of mTOR, and higher p62 levels (Figs. 5B, 5D). Of note, the number of rhodopsin-positive POSs in the *Mfp2^−/−^* RPE on the control diet normalized to WT levels at 8 weeks, most likely because there were no POSs left to be digested due to the completely degenerated retina (Fig. 5B).

Taken together, these results suggest that the depletion of DHA in the *Mfp2^−/−^* retina on the control diet contributed to the lysosomal dysfunction in the RPE at an early age. Furthermore, the temporary rescue of this endolysosomal system could play a role in the delay of RPE dedifferentiation onset in the DHA-supplemented *Mfp2^−/−^* mice.

## Discussion

In this manuscript, we established that the early retinal phenotype of *Mfp2^−/−^* mice is inflicted by an impaired systemic supply of DHA. On the other hand, the retinopathy at later ages is most likely due to the inability of the RPE to handle the VLC-PUFA–containing POSs as a result of impaired peroxisomal β-oxidation.^25^ Altogether, this research provides a better understanding of the factors causing the retinopathy in peroxisome-deficient patients and offers insight into the distinct roles of DHA in the neural retina and RPE cells.

First, in this manuscript we have confirmed that adequate levels of retinal DHA are required for normal postnatal photoreceptor development and function. Furthermore, the rescue of the early *Mfp2^−/−^* photoreceptor phenotype via increasing the systemic supply of DHA highlights the involvement of DHA in the early retinal degeneration in *Mfp2^−/−^* mice. These conclusions are in line with other mouse models with a genetic defect in the acquisition of retinal DHA, showing a similar retinal phenotype.^4^ However, the molecular details regarding how DHA influences photoreceptor homeostasis are still not fully understood. Although the role of DHA in photoreceptor biogenesis and phototransduction has been well studied, its role in oxidative stress is controversial.^2^^,^^4^^,^^51^^,^^52^ On the one hand, it has been shown that, under uncompensated oxidative stress, RPE cells convert DHA to the protective lipid mediators neuroprotectin D1 (NPD1), resolvins, protectins, and maresins.^53^ On the other hand, the high content of double bonds in this PUFA predisposes to lipid peroxidation, which can be deleterious for photoreceptors.^54^^–^^57^ It was suggested that the impact of DHA may depend on the circumstances such as the level of oxidative stress.^58^ Interestingly, targeted metabolome analysis of *Mfp2^−/−^* RPE revealed that levels of redox metabolites (e.g., methionine sulfoxide, reduced glutathione, oxidized glutathione, NAD[H]) were unchanged at 3 weeks.^25^ In addition, levels of antioxidant enzymes (e.g., superoxide dismutase 2) and 4-hydroxynonenal (4-HNE) immunoreactivity were unaltered. In agreement, despite several attempts, we failed to reliably measure the protective lipid mediators in (DHA-supplemented) *Mfp2^−/−^* mice. However, we cannot exclude that, upon DHA supplementation, more NPD1 was generated in the *Mfp2^−/−^* retina, thereby contributing to delay of the retinal degeneration. To investigate a potential protective role of NPD1, *Mfp2^−/−^* mice could be supplemented with this mediator.

Although no statistics could be performed, the lipidome analysis of the neural retina of DHA-supplemented *Mfp2^−/−^* mice revealed a striking accumulation of VLC-PUFA–containing phospholipid species already at the age of 4 weeks. Interestingly, this accumulation was more pronounced in the DHA-supplemented *Mfp2^−/−^* mice compared to *Mfp2^−/−^* mice on the control diet. This confirms our previous findings that the vast accretion of VLC-PUFAs does not initiate the early retinopathy in *Mfp2^−/−^* mice.^26^ However, the inability to process the VLC-PUFAs due to dysfunctional peroxisomal β-oxidation in the RPE could cause the VLC-PUFAs to reach a toxic threshold, thereby contributing to the destruction of the retina at 16 weeks.

Remarkably, VLC-PUFAs were differentially distributed in the *Mfp2^−/−^* retina on the DHA diet. PC-containing VLC-PUFAs (≥C38) were further increased by the diet in *Mfp2^−/−^* retina, whereas TG species containing these VLC-PUFAs were partially reduced, coinciding with lowered lipid droplet accumulation in both the RPE and neural retina. So far, the mechanism by which DHA affects the storage of fatty acids remains unknown. It would be interesting to track the fate of PUFAs and gain insight into the composition of the lipid droplets in the (DHA-supplemented) *Mfp2^−/−^* retinas by applying the recently developed techniques (1) matrix-assisted laser desorption/ionization (MALDI)–mass spectrometry imaging (MSI),^59^^–^^62^ (2) colocalization studies of injected photoreactive lipid probes^63^ and lipid droplets visualized with superresolution microscopy, or (3) in vivo administration of radiolabeled DHA combined with spatial lipidomics.^64^

The most intriguing and unexpected finding in this study was that DHA temporarily improved RPE homeostasis. This seems indeed to be in contradiction with the cell-autonomous role of MFP2 in the RPE, shown by using *Best1-Mfp2^−/−^* mice.^25^ These mice presented with very early-onset RPE cellular anomalies, including lipid droplet accumulations, lysosomal dysfunction, accumulation of undigested POS, RPE dedifferentiation (consisting of loss of hexagonal shape, RPE depolarization, ablation of visual cycle proteins, and RPE protrusions), and prolonged mTOR activation.

The normal lysosomal function and reduced lipid droplet accumulation in the DHA-supplemented *Mfp2^−/−^* RPE at 4 weeks, despite a 20- to 200-fold accumulation of VLC-PUFA–containing lipid species in their neural retina, indicated that the *Mfp2^−/−^* RPE supplemented with DHA is somehow able to handle the VLC-PUFA–containing POSs. Interestingly, POS accumulation in the RPE was also observed in other DHA-deficient mouse models (e.g., *AdipoR1^−/−^*^65^ and *Mfsd2a^−/−^* mice^66^), suggesting that DHA might play a role in the phagocytosis process. Perhaps DHA influences the membrane characteristics, thereby affecting the breakdown of POS phagosomes. However, despite continuous DHA supplementation, the lysosomal function subsequently declined. To gain insight into the temporary rescue of lysosomal functioning, it will be important to determine the sequence of impaired lysosomal function versus accumulation of lipids in the *Mfp2^−/−^* RPE. In vitro studies supplementing normal POS versus DHA-deprived POS to healthy and *Mfp2^−/−^* RPE cells could shed some light on the mechanisms of RPE disruptions and the role of DHA in POS phagocytosis. Nevertheless, it remains unsolved how the DHA-supplemented *Mfp2^−/−^* RPE is able to handle the VLC-PUFAs present in the POS.

The transient rescue of visual cycle genes was remarkable and provided several essential insights. First, the normal levels of visual cycle genes at 4 weeks and suppression at 8 weeks in DHA-supplemented *Mfp2^−/−^* mice correlated with the respective normal and impaired scotopic a-wave responses at these ages. Because the visual cycle genes play a crucial role in the phototransduction, it is plausible that alterations in their levels impaired the rod photoreceptor responses of the DHA-supplemented *Mfp2^−/−^* mice at 8 weeks. Second, both RPE65 depletion^67^ and dedifferentiation of the RPE are known causes of retinal degeneration,^68^ suggesting that these factors contributed to the retinopathy at 16 weeks in the DHA-supplemented *Mfp2^−/−^* mice. However, it remains to be determined what drives the reduction of the visual cycle genes. Importantly, we excluded the notion that changes in the visual cycle genes were due to a developmental problem, as RPE65 levels were unchanged on both protein and RNA levels in 3-week-old *Mfp2^−/−^* mice.^25^ Other factors that are known to regulate mRNA levels of the visual cycle genes include the transcription factors *Sox9* and *Otx2*^50^ and retinoic acid.^69^ However, mRNA levels of *Sox9* and *Otx2* were unaltered at 4 weeks in the *Mfp2^−/−^* RPE. It remains to be investigated whether retinoid levels are changed in *Mfp2^−/−^* retinas. Furthermore, it is still unclear if the reduction in visual cycle genes is either the first sign of RPE dedifferentiation or an independent event. As loss of functional RPE65 or lecithin retinol acyltransferase (LRAT) in mice does not cause RPE dedifferentiation,^67^^,^^70^^–^^76^ it seems that levels of the visual cycle genes are independently regulated from the dedifferentiation process.

The short-term rescue of the RPE phenotype in *Mfp2^−/−^* mice upon DHA supplementation is supported by other mouse models with a genetic defect in retinal PUFA acquisition, in which several RPE abnormalities were reported.^4^ However, it remains to be determined whether the RPE phenotype is due to a primary deficiency of DHA levels in the RPE or a secondary effect of neural retina degeneration. Interestingly, comparison of the retinal phenotype of *Crx-Mfp2^−/−^*^26^ and *Best1-Mfp2^−/−^* mice^25^ indicated that loss of MFP2 from the RPE, and not from photoreceptors, is detrimental for the retina. Taken together, it seems worthwhile to further explore the role of DHA in the RPE.

What are the implications of this research for therapeutic approaches for peroxisome-deficient patients? Major drawbacks with regard to retinal research in peroxisomal disorders include the translatability of the findings from the mouse models to patients due to (1) the lack of reports on the histopathological changes and lipid content of the retina of peroxisome-deficient patients; (2) the promising, but inconclusive, results of clinical trials with DHA supplementation for peroxisome-deficient patients^77^^,^^78^; and (3) differences in cone density between human and mice retinas. This hinders drawing conclusions regarding the causative factors underlying the retinopathy in peroxisome-deficient patients and thus the ability to recommend a treatment option. Nevertheless, the present data imply that sole DHA supplementation will not be able to prevent the retinopathy in patients with peroxisome deficiencies. Therefore, a combination approach of DHA supplementation to normalize the systemic DHA supply together with local viral delivery of the missing gene (as already reported for *Pex1*-mutant mice)^79^ in order to process the (VLC-)PUFAs in the RPE and neural retina seems plausible. This dual approach will be necessary to prevent both the early onset (i.e., reduced retinal DHA levels, due to impaired systemic supply) and late retinopathy (i.e., impaired handling of the VLC-PUFA–containing POS due to the cell-autonomous role of MFP2 in the RPE).

## Supplementary Material

Supplement 1
