# Supplementary material for: DHA Shortage Causes the Early Degeneration of Photoreceptors and RPE in Mice With Peroxisomal β-Oxidation Deficiency
Source: Invest Ophthalmol Vis Sci. 2023 Nov 7;64(14):10. doi: 10.1167/iovs.64.14.10 (PMC10631513; doi:10.1167/iovs.64.14.10)
Supplement: Supplement 1 [file iovs-64-14-10_s001.pdf]

Supplementary figures

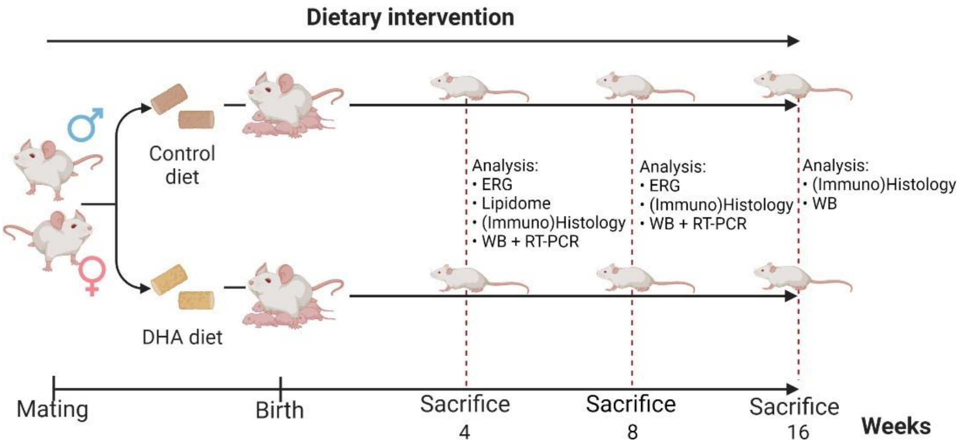

Figure S.1. DHA supplementation to Mfp2$^{-/-}$ mice. *At the moment of mating, breeding pairs were put on either a control or DHA diet. Consequently, the Mfp2$^{-/-}$ pups and controls already receive the diet during the gestation and lactation period. After weaning (±P21), offspring received the diet until sacrifice. Figure created with Biorender.com.*

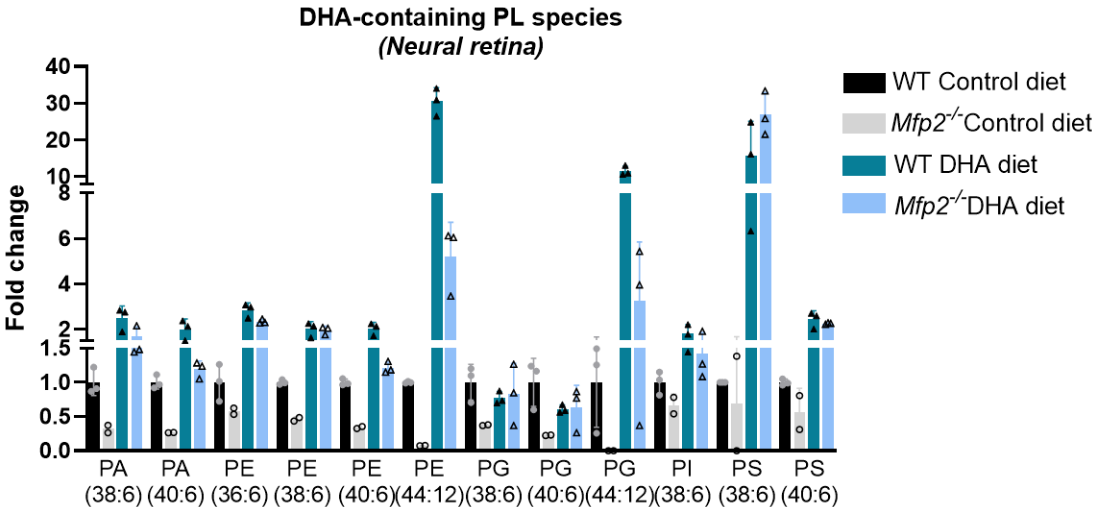

Figure S.2. DHA-containing phospholipid species*. Lipidome analysis represented as fold change for phospholipids (PL) most likely containing one or two DHA moieties (based on the presence of 6 or 12 double bonds). N=2-3/group. No statistical test was performed, but individual data points are shown. Error bars indicate SD. PA—phosphatidic acid; PE—phosphatidylethanolamine; PG—phosphatidylglycerol; PI—phosphatidylinositol; PS—phosphatidylserine.*

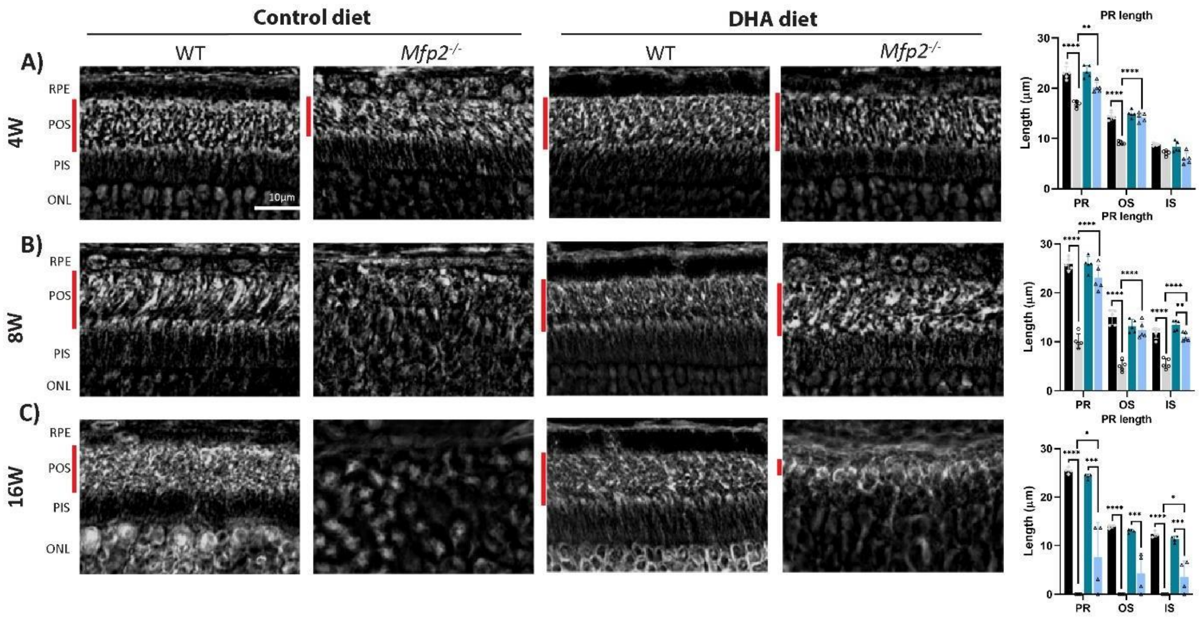

Figure S.3. Delayed outer segment shortening in Mfp2$^{-/-}$ mice on DHA diet. *Measurement of photoreceptor layers on phase contrast microscopy images in 4- **(A)**, 8- **(B),** and 16-week-old **(C)** mice. Red bars indicate POS length. N=4-5/group. Statistical difference based on multiple one-way ANOVA. Error bars indicate SD. RPE—retinal pigment epithelium; POS—photoreceptor outer segments; PIS—photoreceptor inner segments; ONL—outer nuclear layer; PR—photoreceptor. * $p < 0.05$, ** $p < 0.01$, *** $p < 0.001$, **** $p < 0.0001$.*

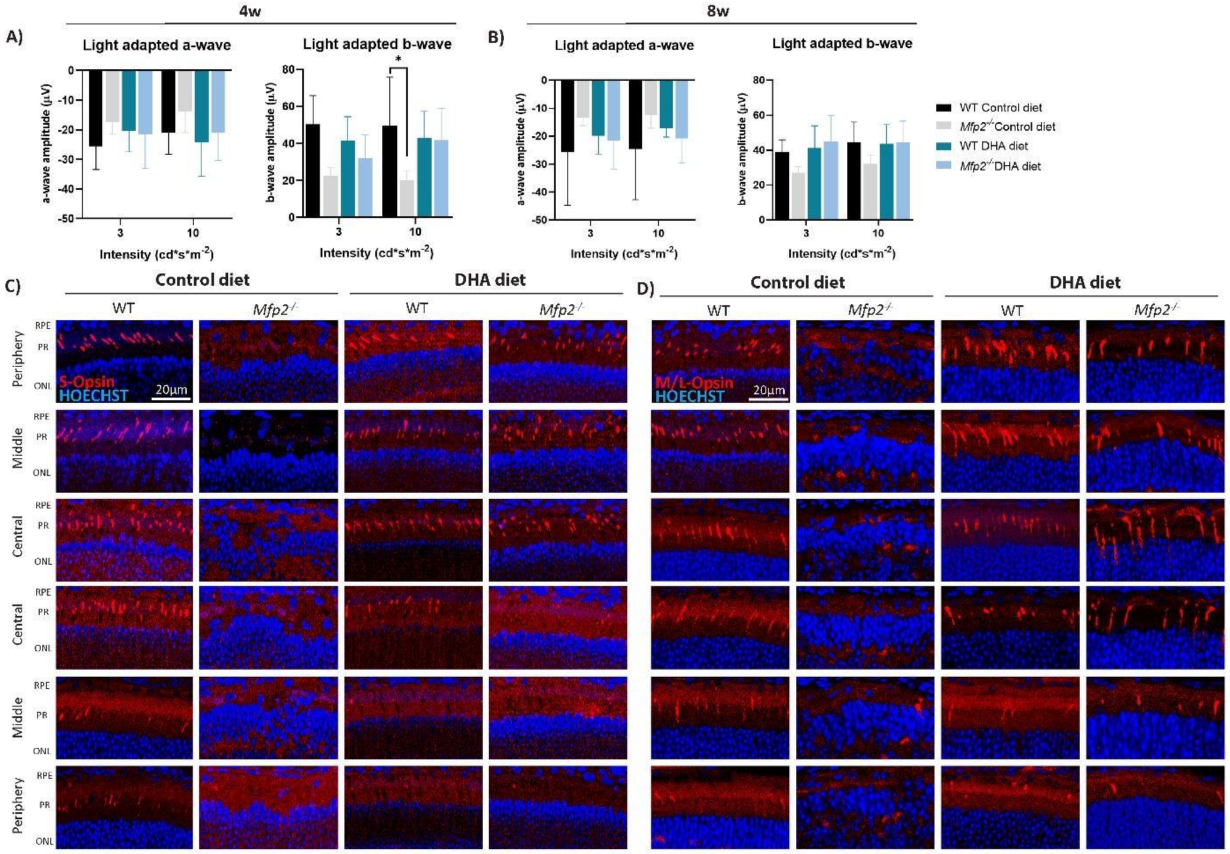

Figure S.4. Cone function and distribution in (DHA-supplemented) Mfp2$^{-/-}$ mice. *(**A**) Light adapted ERG responses from 4- and **(B)** 8-week-old mice. Statistical difference based on multiple two-way ANOVA. Error bars indicate SD. **(C)** Cone-specific stainings on 8-week-old retinal sections visualized with S-opsin (red) and **(D)** M/L-opsin (red). Pictures are taken in 6 different regions on both sides (nasal and temporal) of the optic nerve head. N=4/group. RPE—retinal pigment epithelium; PR—photoreceptor; ONL—outer nuclear layer; $*p < 0.05$.*

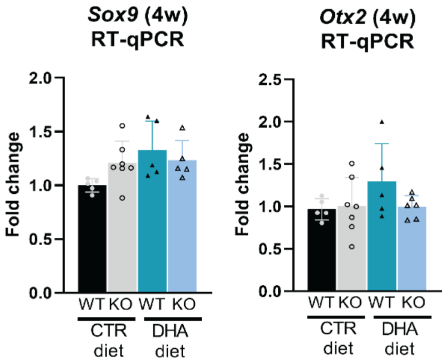

Figure S.5. RT-qPCR for transcription factors regulating visual cycle protein expression (4w). *N=5-8/group. Statistical difference based on multiple one-way ANOVA. Error bars indicate SD. Otx2—orthodenticle homeobox 2; Sox9—SRY-box transcription factor 9.*

SUPPLEMENTARY TABLES

Supplementary Table 1. *List of primers used for PCR genotyping.*

Gene	Forward	Reverse
Mfp2+	5′ ACGCAGGCGCACTTGGAACCACCG 3′	5′ GTGACCAAGACCACACGCCCG 3′
Mfp2-	5′ TGGCAAGTGGAATACCAACACCCT 3′	5′ CGCATCGCCTTCTATCGCCTTCTTG 3′
Pde6 WT	5’ ACCTGCATGTGAACCCAGTATTCTATC 3’	5’ CTACAGCCCCTCTCCAAGGTTTATAG 3’
Pde6 mutant	5’ CTACAGCCCCTCTCCAAGGTTTATAG 3’	5’ AAGCTAGCTGCAGTAACGCCATTT 3’

Mfp2—multifunctional protein 2; Pde6—phosphodiesterase 6.

Supplementary Table 2. *List of used primary antibodies for immunohistochemical stainings and immunoblotting.*

Target of primary antibody	Host	Dilution	Application	Supplier/Reference
Ezrin	Rabbit	1/200	NDF section	Cell signaling technology (3145)
GFAP	Rabbit	1/10,000	NDF section	Dako (Z0334)
IBA1	Rabbit	1/500	NDF section	Wako (019-19741)
M/L-Opsin	Rabbit	1/100	NDF section	Millipore (AB5405)
S-Opsin	Rabbit	1/100	NDF section	NOVUS (NBP1-20194)
p62	Rabbit	1/500	Immunoblotting	Abcam(ab109012)
PLIN2	Rabbit	1/1,000<br>1/500	NDF section<br>Immunoblotting	NOVUS (NB110-40877)
Peanut agglutinin lectin	-	1/100	NDF section	Vector Laboratories (FL-1071)
P-s6	Rabbit	1/500	Immunoblotting	Cell signaling technology (4858)
Rhodopsin (1D4)	Mouse	1/1,000<br>1/20,000	NDF section<br>Immunoblotting	Millipore (MAB5356)
Rhodopsin (B630)	Mouse	1/1,000	NDF section	Novus (NBP2-25160)
RPE65	Mouse	1/100<br>1/1,000	NDF section<br>Immunoblotting	Invitrogen (MA1-16578)
s6	Rabbit	1/500	Immunoblotting	Cell signaling technology (2217)
Vinculin	Mouse	1/2,000	Immunoblotting	Sigma (V9131)
ZO-1	Rabbit	1/100	RPE flatmount	Invitrogen (61-7300)

GFAP—glial fibrillary acidic protein; IBA1—ionized calcium-binding adapter molecule 1; NDF—new davidson’s fixative; PLIN2—perilipin 2; RPE65—65 kDa retinoid isomerohydrolase; ZO-1—zonula occludens-1.

Supplementary Table 3. *List of primers used for RT-qPCR.*

Gene	Forward	Reverse
Actb	5’ ATTGGCAACGAGCGGTT 3’	5’ AGGTCTTTACGGATGTCAACG 3’
Lrat	5’ GCGAACACTTTGTGACTTACTG 3’	5’ AAGACAGCCGAAGCAAGAC 3’
Otx2	5’ AAATCAACTTGCCAGAATCCAG 3’	5’ TTGTTCTGACCTCCATTCTGC 3’
Rdh5	5’ GTCAACATCACCAGTGTCTTG 3’	5’ GAGACTTGTACTCCGAACGG 3’
Rho	5‘ ACCCTCTACACATCACTCCAT 3‘	5’ CTTGCAGACCACCACGTAG 3‘
Rpe65	5‘ TGACAAGGTCGACACAGGCAGAAA 3‘	5’ AAATTCAAAGGCTTGACGAGGCCC 3’
Sox9	5’ CGACCCATGAACGCCTT 3’	5’ GTCTCTTCTGCTCTCGTTC 3’

Actb—β-actin; Lrat—lecithin retinol acyltransferase; Otx2—orthodenticle homeobox 2; Rdh5—retinol dehydrogenase 5; Rho—rhodopsin; Rpe65—65 kDa retinoid isomerohydrolase; Sox9—SRY-box transcription factor 9.

Supplementary Table 4. *Lipid levels in plasma (µmol/L).**

Lipids	WT Control diet	*Mfp2*$^{-/-}$ Control diet	WT DHA diet	*Mfp2*$^{-/-}$ DHA diet
C16:0	2538.9 ±451.8	1704.3 ±352.0	2487.4 ±415.5	1099.2 ±412.6
C18:0	1169.3 ±161.0	883.3 ±125.7	1051.7 ±343.7	683.4 ±263.0
C18:1n-9	1163.7 ±322.1	770.2 ±609.0	960.6 ±276.8	284.9 ±138.4
C18:2n-6	3480.6 ±848.8	2459.8 ±70.8	4091.0 ±1894.1	1975.9 ±839.3
C18:3n-3 (ALA)	4.4 ±2.2	1.6 ±0.3	63.4 ±46.7	6.7 ±2.8
C18:3n-6	41.2 ±16.4	23.8 ±7.1	46.2 ±14.1	15.8 ±2.1
C20:0	19.7 ±2.0	16.7 ±2.0	20.0 ±10.7	15.8 ±3.6
C20:4n-6 (AA)	1551.4 ±447.9	1103.5 ±122.9	1092.1 ±261.4	746.9 ±244.0
C20:5n-3 (EPA)	16.4 ±7.3	8.0 ±2.7	36.7 ±7.1	8.8 ±4.3
C22:0	22.4 ±4.6	16.0 ±1.8	22.2 ±5.4	15.3 ±3.2
C22:4n-6	57.1 ±18.8	23.4 ±6.6	24.3 ±8.3	10.5 ±1.1
C22:5n-3 (DPA)	3.0 ±1.6	0.8 ±0.4	30.6 ±6.0	8.5 ±4.6
C22:5n-6	235.2 ±44.0	197.4 ±34.9	15.9 ±6.8	18.4 ± 3.7
C22:6n-3 (DHA)	48.0 ±10.6	17.7 ±6.6	391.8 ±26.2	225.5 ±35.2
C24:0	15.9 ±2.3	35.9 ±3.5	15.0 ±2.9	32.0 ±4.72

*AA—arachidonic acid; ALA—α-linolenic acid; DHA—docosahexaenoic acid; DPA—docosapentaenoic acid; EPA—eicosapentaenoic acid. *Only the relevant fatty acids are shown.*
